# Effects of Chinese Medicine as Adjunct Medication for Adjuvant Chemotherapy Treatments of Non-Small Cell Lung Cancer Patients

**DOI:** 10.1038/srep46524

**Published:** 2017-04-24

**Authors:** Lijing Jiao, Changsheng Dong, Jiaxiang Liu, Zhiwei Chen, Lei Zhang, Jianfang Xu, Xiaoyong Shen, Jiaming Che, Yi Yang, Hai Huang, Hegen Li, Jianli Sun, Yi Jiang, Zhujun Mao, Peiqi Chen, Yabin Gong, Xiaolin Jin, Ling Xu

**Affiliations:** 1 Department of Oncology, Longhua Hospital, Shanghai University of Traditional Chinese Medicine, Shanghai 200032, China; 2 Tumor Institute of Traditional Chinese Medicine, Shanghai Research Institute of Traditional Chinese Medicine, Shanghai 200032, China; 3 Lung Tumor Clinical Medical Center, Shanghai Chest Hospital, Shanghai Jiaotong University, Shanghai 200030, China; 4 Department of Thoracic surgery, Shanghai Pulmonary Hospital, Tongji University, Shanghai 200433, China; 5 Departmentof Oncology, Shanghai Pulmonary Hospital, Tongji University, Shanghai 200433, China; 6 Department of Thoracic Surgery, Huadong Hospital, Fudan University, Shanghai 200040, China; 7 Department of Thoracic Surgery, Ruijin Hospital, Shanghai Jiaotong University, Shanghai 200025, China; 8 Department of Thoracic Surgery, Shanghai Sixth People’s Hospital, Shanghai Jiaotong University, Shanghai 200030, China; 9 Department of Pneumology, Changzheng Hospital, The Second Military Medical University, Shanghai 200003, China; 10 Department of Oncology, Yueyang Hospital of Integrated Traditional Chinese and Western Medicine, Shanghai University of Traditional Chinese Medicine, Shanghai 200437, China

## Abstract

The aim was to evaluate the effects of traditional Chinese medicine (TCM) as a combination medication with adjuvant chemotherapy on postoperative early stage non-small cell lung cancer (NSCLC) patients. The 314 patients with completely resected stage IB, II or IIIA cancers were assigned into vinorelbine plus cisplatin/carboplatin (NP/NC) (control, *n* = 158) and NP/NC with additional TCM (intervention, *n* = 156) groups. The primary endpoint was QOL scores; secondary endpoints were the toxicity and safety of the regimens. The NP/NC regimen caused mild (grade 1 or 2) non-hematologic toxic effects in the patients comprising vomiting (43.6%), fatigue (36.9%), pain (23%), dry mouth (27.6%) and diarrhea (7.9%). The incidence of adverse events was significantly lower in the intervention group than in the control group (0.57% *vs* 4.02%, *P* = 0.037). Transient severe (grade 3 or 4) hematological toxic effects occurred less often (hemoglobin reduction (11.9 *vs* 22.5 percent) and total bilirubin increased (to 42.1 vs 46.2%) in the intervention compared to the control group during the 2nd chemotherapy cycle. When combined with adjuvant chemotherapy, TCM led to partial relief of symptoms in addition to a reduction of side-effects and adverse events caused by the NP/NC regimens.

Lung cancer is one of the malignancies that has a high incidence and mortality rate. Approximately 75–85% of all lung cancers are Non-Small Cell Lung Cancers (NSCLC), including squamous carcinoma, adenocarcinoma and large cell carcinoma^1^. Currently, surgical resection is the primary option for NSCLC in the early to middle stages. Stages Ia, II and part of stage IIIa of NSCLC can be completely excised by surgery, with the postoperative 5-year survival rate being 77% for Ia NSCLC, but only 23% for IIIa NSCLC^2^. Local recurrence and distant metastasis are the main factors for prognosis. It has become standard procedure that Ib-IIIa NSCLC patients receive adjuvant chemotherapy after radical surgery, which led to a 5-year survival rate increase from 54% to 69%, but the toxic effects on the quality of life of patients are still a concern for adjuvant medication and might lead to decreased compliance rates during the required four cycles of chemotherapy^3^.

Qi in TCM mainly means full of function and makes up the body as well as maintains life activities. All vital substances in the body are transformed by constant motion and changes of qi^4^. Traditional Chinese medicine (TCM) includes mainly herbal medications and in its holistic approach TCM believes that a tumor is caused by shortness of the healthy qi, leading to organ dysfunction and the stagnation of qi and blood^5^. Then, pathological products such as dirty sputum and bruises emerge and accumulate into tumor masses. Diarrhea, hair loss, insomnia and pain symptoms arise as the tumor develops or when chemotherapeutic drugs are administered, which further reduces the healthy qi. Therefore, the key point of TCM cancer treatment is to strengthen a healthy qi and eliminate pathogenic factors. However, after analysis of TCM herb compounds used for cancer therapy, the effects of TCM medication were mainly attributed to the inhibition of cell proliferation and the induction of apoptosis via various mechanisms, as well as inhibition of angiogenesis and regulation of the immune system^5^,^6^. Since western medicine chemotherapy regimens have been adopted in China, TCM is mainly applied in clinical practice in combination with adjuvant chemotherapy and the formulas have been extended to cope with chemotherapy-related side effects in addition to prevention of cancer recurrence and metastasis as well as prolonging the survival time of postoperative cancer patients^7^,^8^,^9^,^10^,^11^,^12^.

In the present study, a perspective randomized, double-blinded clinical trial was designed and conducted to evaluate the effects of four TCM formulations covering three different syndromes as adjunct medications, with a NP/NC regimen on clinical symptoms and QOL of postoperative NSCLC patients in comparison to a NP/NC regimen alone. The findings should help to improve adjuvant regimens for NSCLC patients after radical excision of their tumors.

## Methods

### Study design and patients

A randomized, controlled and double-blinded clinical study procedure was performed on 314 Ib-IIIa NSCLC patients enrolled in 7 centers in China. The trial was registered at ClinicalTrials.gov (Number: NCT01441752; Date: July 14, 2011) and enrolment has been completed; patients are still in follow-up. Eligibility criteria included the following: completely resected stage Ib-IIIa NSCLC, ages 18–75 years, An Eastern Cooperative Oncology Group performance status (ECOG PS) scale of 0–2 and adequate hematological, biochemical and organ functions. Chemotherapy was started within 6 weeks after surgery and consisted of 4 cycles of cisplatin/carboplatin and vinorelbine. All data collection and analyses was carried out in Longhua hospital. The primary aim of the study was to evaluate the effectiveness of TCM on QOL for postoperative NSCLC patients treated with adjuvant chemotherapy, and the secondary aim was to evaluate any beneficial effects of TCM on side effects caused by chemotherapy.

Our study was performed in accordance with the Declaration of Helsinki regarding the ethical principles for medical research involving human subjects. The protocol was approved by the ethics boards of Longhua Hospital. All participants signed informed consent forms.

### Randomization

Within six weeks of surgery, eligible patients were randomly assigned in a 1:1 ratio to an adjuvant vinorelbine plus cisplatin/carboplatin (NP/NC) control group (*n* = 158) or a NP/NC plus TCM intervention (*n* = 156) group. Central randomization was implemented by a clinical research organization (CRO) (Shanghai Clinical Research Center, Shanghai, China) via the internet; patients were stratified according to the clinical stage of their cancer (Ib *vs* IIa *vs* IIb *vs* IIIa), histological subtype (adenocarcinoma *vs* squamous carcinoma *vs* glands squamous carcinoma *vs* large cell carcinoma *vs* other types of NSCLC), as well as gender, QOL-LC43 scores, and the center.

### Medication

The NP regimen consisted of 75 mg of cisplatin per square meter of body-surface area on day 1 and 25 mg of vinorelbine per square meter on days 1 and 8 weekly, every 4 weeks for 4 cycles.

The NC regimen included carboplatin (AUC (area under the curve) = 5) on day 1 and 25 mg of vinorelbine per square meter on days 1 and 8 weekly every 4 weeks for 4 cycles.

Based on the *Chinese Medicine New Medicine Clinical Practice Guideline* (Trial Implementation) (published by China Medical Science Press in 2002) and *Shanghai Chinese Medicine Routine Practice* (written by Shanghai Municipal Commission of Health and Family Planning), combined with years of clinical observation and experience in our department, 3 basic syndromes were summarized for lung cancer, and they were determined by two senior physician/deputy chief physicians. Patients with at least 2 main symptoms and 1 secondary symptom could be diagnosed thus:

1. *Qi syndrome deficiency*Main symptoms: cough, large amount of sputum, loss of appetite, fatigue and weakness, pale and bulgy tongue. Secondary symptoms: spontaneous sweat, unshaped stool, thin superficial and smooth pulse.
2. *Yin syndrome deficiency*Main symptoms: cough, small amount of sputum, dried mouth, red tongue. Secondary symptoms: night sweats, insomnia, low fever, thready pulse, rapid pulse.
3. Qi and yin syndrome deficiencyMain symptoms: cough, small amount of sputum, fatigue and weakness, dried mouth without polydipsia. Secondary symptoms: spontaneous sweat, night sweat, reddish tongue or tongue with teeth imprints, thready and weak pulse.

All medicines used were sourced from the same producing area and the same batch (Jiangyin Tian Jiang Pharmaceutical Co., Ltd produced the Chinese medicine granules and placebos) and were purchased as ready to use granules.

Chinese medicine applications were started on the first day after chemotherapy, but not the day chemotherapy drugs were applied. The medicine was dissolved into 160 mL of warm water for drinking at 9:30 a.m. and 15:00 p.m. every day until the end of the chemotherapy. All procedures were supervised by clinical research pharmacists. According to the 4 formulas of the TCM, we fabricated 4 kinds of oral placebos of matched weight, color, smell, taste and packaging as the TCM medicine.

1. *Formula I* (including qi and warming yang granules)The ingredients were Radix astragalus, which ameliorates exercise-induced fatigue and chronic fatigue syndrome^13^,^14^, Codonopsis pilosula with immune modulatory^15^ anti-tumor^16^ and anti-gut damage^17^ effects, Atractylodes macrocephala, which has been reported to inhibit allergic diarrhea stimulation of the Th1-type immune responses^18^, Poria cocos, an antioxidant^19^, Trigonella foenum-graecum, an antioxidant^20^, which also enhances endurance by facilitating increased utilization of fatty acids as an energy source^21^ and ameliorates various impairments associated with physical fatigue^22^ as well as Psoralea corylifolia, which is used as an antidepressant by inhibiting monoamine oxidase A and B (MAO-A and MAO-B) activity^23^ (see supplementary Table 1).
2. *Formula II* (nourishing yin and promoting the secretion of body fluid granules):The ingredients were Radix asparagus, Radix adenophorae, which suppress the development of inflammation and decreases airway damage by suppressing T cell activity, eosinophilia and bronchial hyper-responsiveness^24^, Glehnia literalis with anti-cancer effects^25^, Ophiopogon japonicus with antioxidant, immunomodulatory and salivary secretion stimulatory effects^26^,^27^ as well as Lilium brownii with antitumor^28^ anti-fatigue effects^29^ and Ligustrum lucidum, which has been reported to inhibit proliferation and induce apoptosis of cancer cells^30^,^31^,^32^,^33^ (see supplementary Table 2).
3. *Formula III* (yin-nourishing and qi-tonifying granules)Formula III was a combination of formula I and formula II.
4. *Formula IV* (detoxifying and resolving masses granules).The ingredients were the herbs with antitumor effects, Prunella vulgaris L.^34^,^35^,^36^, Arisaema heterophyllum Blume^37^, Rhizoma amorphophalli^38^, Cremastra appendiculata^39^, Seaweed^40^,^41^, Fructus trichosanthis^42^, Ranunculus ternatus^43^,^44^, Euphorbia helioscopia L.^45^, Salvia chinensis Benth^46^, Paris polyphylla^47^ as well as Selaginella doederleinii Hieron with antitumor, anti-inflammatory, anti-oxidant, anti-fungal and anti-virus activity^48^, oyster shell with sedative effects^49^ and Jujube date which has been reported to upregulate erythropoietin expression^50^ and has antioxidative, antitumor^51^ and anti-fatigue^52^ properties (see supplementary Table 3).

The 4 formulas were applied according to the following regimens:

1. *Formula I + Formula IV:* Indications for patients with qi deficiency syndrome.
2. *Formula II + Formula IV:* Indications for patients with yin deficiency syndrome.
3. *Formula III + Formula IV:* Indications for patients with qi and yin deficiency syndrome.

### Assessments

A modified version of the European Organization for Research and Treatment of Cancer (EORTC) quality of life questionnaire (QLQ-C30) was used to evaluate QOL changes (EORTC QLQ-LC43), which comprised 15 questions derived from the EORTC QLQ-C30 and 10 questions derived from the EORTC QLQ-LC13 questionnaires^53^. The data from every week during each cycle of chemotherapy until disease progression were collected. A high functional area score and a low symptom domain score indicated an improved quality of life. A change in score of 10 points from baseline was defined as significant in functional domain and symptom QOL determinations on the basis of previous studies^54^,^55^,^56^ and published guidelines from the National Cancer Institute of Canada clinical trial group^57^.

All adverse and serious adverse events were recorded. Adverse events were defined by grade according to the National Cancer Institute Common Terminology Criteria for Adverse Events version 3 (NCI CTC AE 3.0), with an adverse event being defined as unanticipated symptoms, physical signs, disease or other body injuries, which might be linked to the administered medicine, yet not necessarily caused by them. All adverse events were coded by the Medical Dictionary for Regulatory Activities 14.0 (MedDRA14.0) and analyzed for each experimental patient group.

### Statistical analysis

SAS software (version 9.2) was used for statistical analysis. Attributes data (QOL scale): a paired *t*-test was used for comparing the average score before and after treatment, and an independent-sample *t*-test to compare the score between groups. According to the homogeneity of variance test, data with a *P*-value not fitting a normal distribution was analyzed using a rank sum test between groups. For variable data (baseline, medicine for adjuvant chemotherapy), a chi-square test was used.

Ordered hierarchical data (NCI-CTC graded adverse events) were analyzed using a rank sum test. Analysis of demography and baseline characteristics was based on the full analysis set. According to the types and characteristics of the different variables, a *t*-test/Wilcoxon rank sum test was used for baseline comparison of the quantitative index and a chi-square/Fisher exact test for the baseline comparison of the qualitative index.

We used a full analysis set (FAS) to analyze the adverse events, which was comprised of all enrolled patients who met the inclusion criteria including lost but not excluded patients. The pre-protocol set (PPS) was composed of enrolled patients who completed the trial according to the protocol for QOL analyses.

According to a previous study, after three-month adjuvant chemotherapy of early lung cancer patients the QOL score assessed by the QLQ-C30 scale decreased by 27% compared with baseline^56^. With an estimated 15% of adjuvant plus TCM therapy patients with no deterioration of QOL and a significance level of α = 0.05, 1-β = 0.90 and a QOL score decline of 12% in the treatment and 27% in the control group after three-months, 198 cases was the necessary sample size in each group after power analysis. Considering a 20% drop-off rate, the research team was expected to observed 480 cases (*n* = 240 cases in each group) for 2 years to compare QOLs of postoperative NSCLC patients treated with chemotherapy with or without adjunctive TCM medication.

## Results

### Patient characteristics and the primary outcome

The study period was from December 14, 2012 until August 1st 2015. As shown in Table 1, 44.23% of the patients had pathological stage IB disease, 23.08% had stage II, and 32.69% had stage III disease and all had an Eastern Cooperative Oncology Group performance status score of 0 to 2; the median age was 59 years; 60.90% were men and 71.79% had adenocarcinomas. There was no statistically significant differences between the groups regarding demographic and baseline characteristics (Table 1).

### Compliance to QOL Assessment

**Figure 1:**
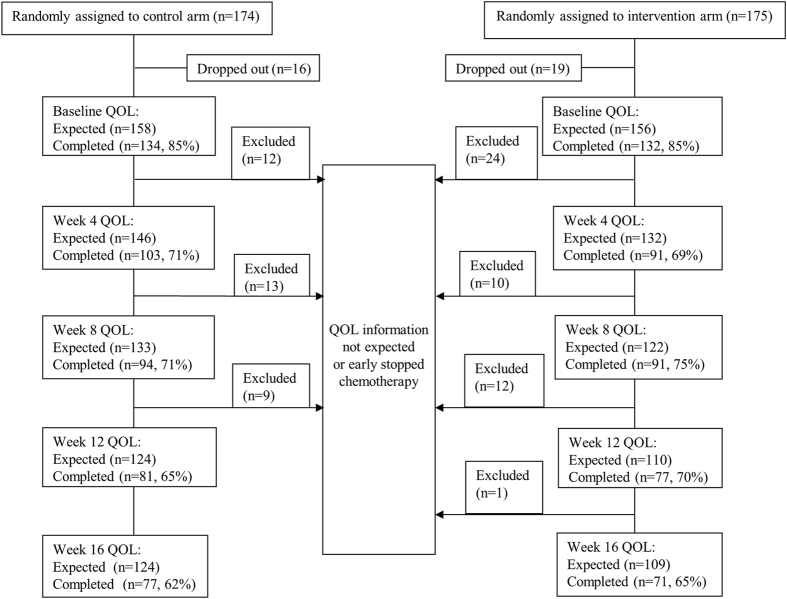
Flow chart of compliance with quality of life (QOL) questionnaire completion.

In our study, the compliance with the QOL assessment is summarized in Fig. 1. In the intervention arm, 134 patients completed the baseline QOL assessment, which represented 85% of all patients from this group. Similar compliance (132 patients; 85% of expected) was noted in the control arm.

In the entire study, 49.35% (77 of 156) patients in the intervention arm and 44.93% (71 of 158) patients in the control arm completed the QOL questionnaire until week 16 of the study.

### Baseline QOL Results

**Figure 2:**
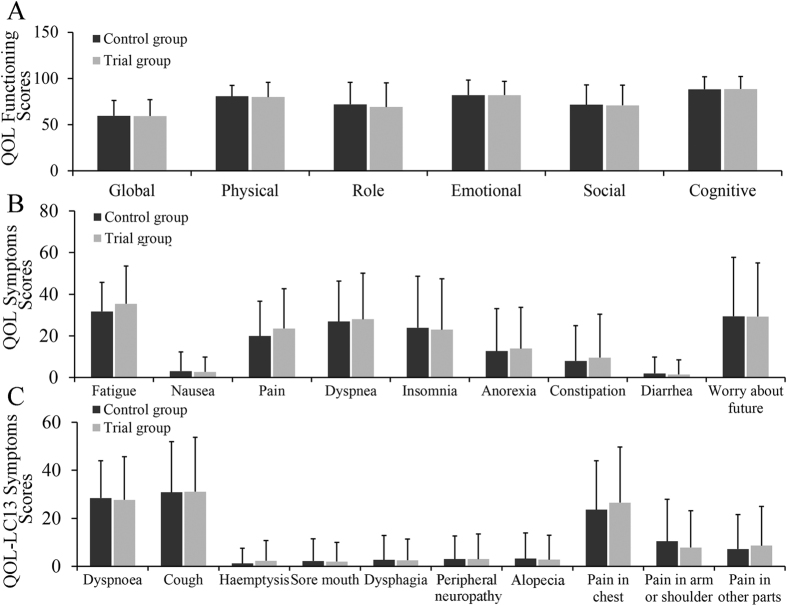
Baseline QOL scores in the two groups. (**A**) Functioning scores, (**B**) Symptom scores, (**C**) Symptom scores.

The two arms had similar baseline QOL scores in all domains and items (Fig. 2).

The most obvious weaknesses were global functioning for early stage postoperative lung cancer patients (mean 59.2 ± 17.9), followed by role functioning (mean 69.1 ± 26.1), social functioning (mean 70.8 ± 21.9), physical functioning (mean 79.8 ± 16), emotional functioning (mean 81.9 ± 15) and cognitive functioning (mean 88.5 ± 13.5). The most common symptoms in single items were fatigue (mean 35.4 ± 18.1), worry about the future (mean 29.3 ± 25.7), dyspnea (mean 28.0 ± 22.1), sleep problems (mean 23 ± 2 4.4) and pain (mean 23.5 ± 19.2). QOL-LC13 scores of the lung cancer-associated symptoms of coughing (mean 31.1 ± 22.6), dyspnea (mean 27.7 ± 18) and pain in the chest (mean 26.5 ± 23.2) were increased.

### QOL in the early postsurgery period (baseline to 4 months)

**Figure 3:**
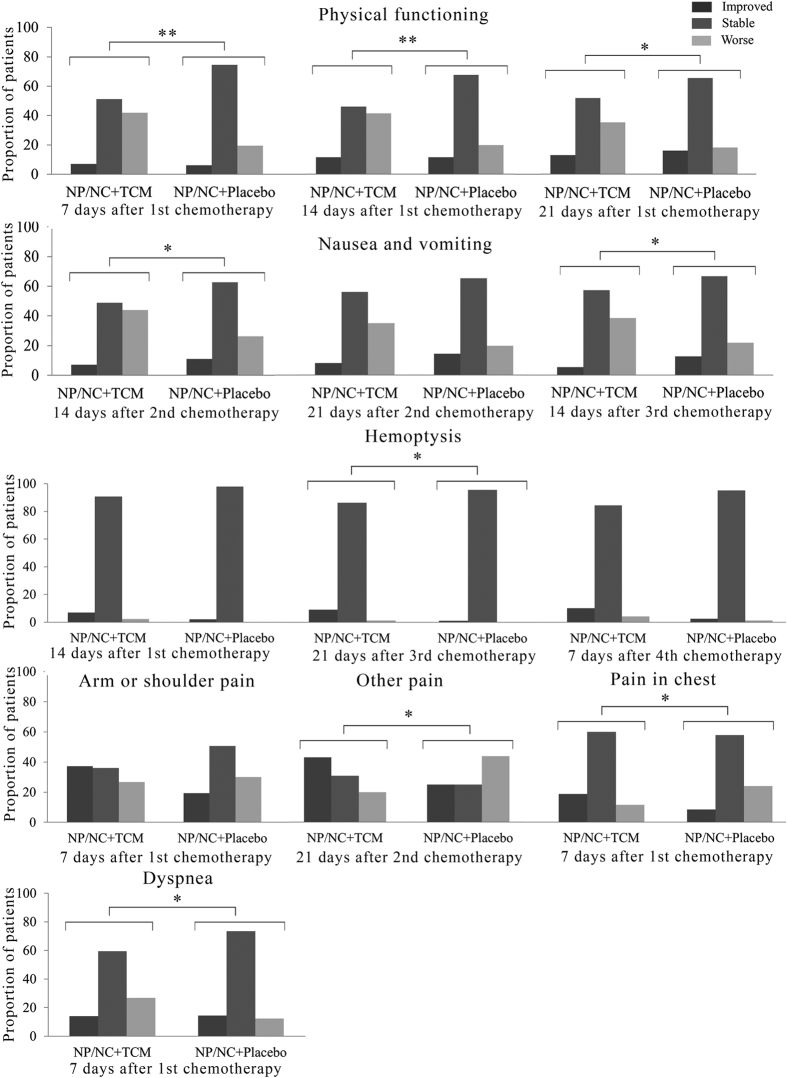
Proportion of patients with improved, stable and worsened quality of life, in several domains and symptoms during the 1^st^, 2^nd^, 3^rd^ and 4^th^ cycle of chemotherapy. *Indicates *P* < 0.05; **Indicates *P*  < 0.005.

Analysis of the proportion of patients who had QOL scores that improved, remained stable or became worse compared with the baseline at each time point after the start of chemotherapy are shown in Fig. 3. Physical functioning was superior at 7 (*P* < 0.01), 14 (*P* < 0.01) and 21 days (*P* < 0.05) after 1^st^ chemotherapy in the control group. In the symptom domains, significantly more patients in the control group experienced improved nausea/vomiting scores at 14 days (*P* < 0.05) after 2^nd^ 21 days (*P* < 0.05) 14 days after the 3^rd^ cycle. For single items, NP/NC chemotherapy plus TCM patients showed fewer symptoms of pain in the chest at 7 days after 1^st^ chemotherapy (*P* < 0.05), hemoptysis at 21 days after 3^rd^ chemotherapy (*P* < 0.05), other pain at 21 days after 2^nd^ chemotherapy (*P* < 0.05), whereas dyspnea was less improved compared to the control group at 7 days after 1^st^ chemotherapy (*P* < 0.05) (Fig. 3).

### QOL after completed chemotherapies

**Figure 4:**
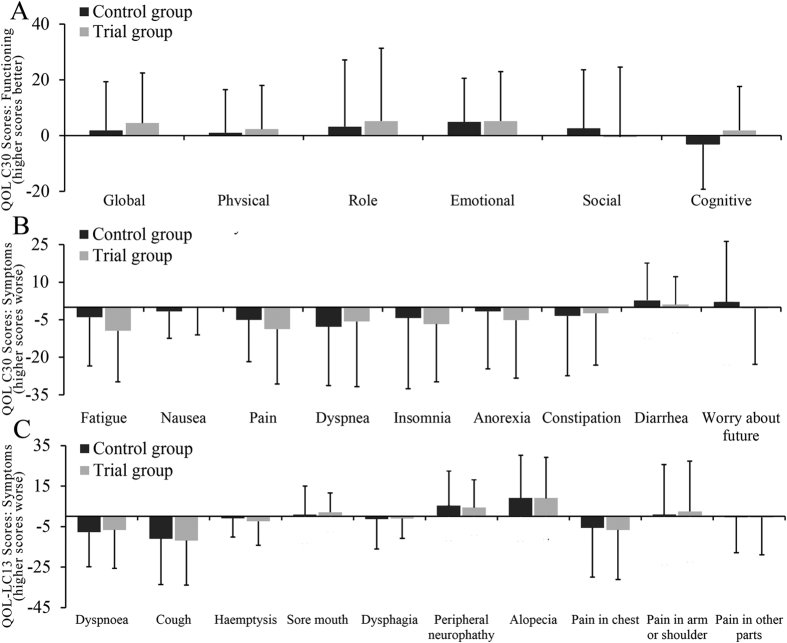
Summary of the changes of QOL before and after treatment. (**A**) Functioning scores, (**B**) Symptom scores, (**C**) Symptom scores.

After completed treatment, in the intervention group, the overall functioning scores beside social functioning (Fig. 4A) were improved, and overall symptom scores such as fatigue, pain, insomnia anorexia (Fig. 4B) as well as cough and chest pain (Fig. 4C) were also decreased, though all without statistical significance. These changes are illustrated in the bars, which depicts the difference scores before and after the 4^th^ cycle of chemotherapy. In Fig. 4B,C, data below zero indicates that the post-therapy scores were lower than the baseline scores which meant an improvement in related symptoms. Positive scores in Fig. 4B,C indicate that the symptoms became worse after the treatment.

### Safety and adverse events

The NP/NC regimens caused mild (grade 1 or 2) non-hematologic toxic effects in patients comprising vomiting (43.6%), fatigue (36.9%), pain (23%), dry mouth (27.6%), anorexia (20%) as well as diarrhea (7.9%), and chemotherapy plus TCM was associated with a significantly lower incidence of adverse events than was chemotherapy alone (0.57% *vs* 4.02%, *P* = 0.037) (Table 2). Tables 3 and 4 summarize the main reported hematological and non-hematological adverse events. After 7 days and 14 days of each chemotherapy cycle, TCM significantly reduced the incidence of non-hematological adverse events of pain (*P* = 0.01), dry mouth (*P* = 0.007, 0.04, 0.037), vomiting (*P* = 0.004), diarrhea (*P* = 0.02, 0.011) and fatigue (*P* = 0.03).

**Figure 5:**
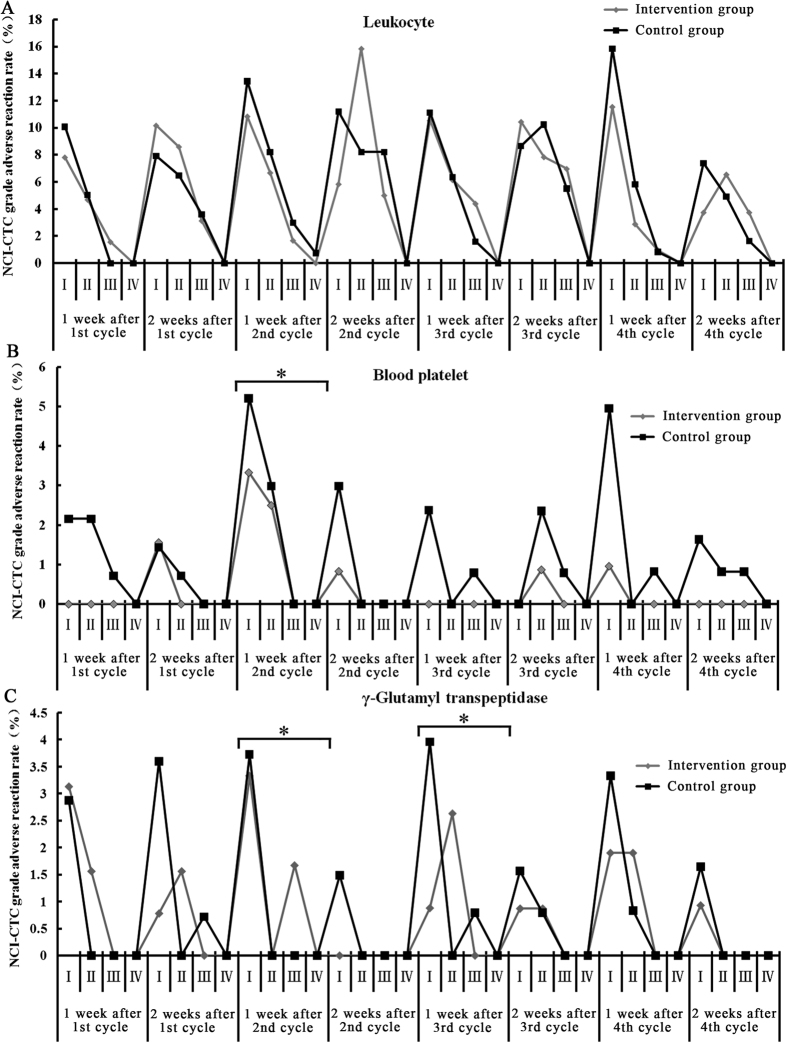
NCI-CTC graded adverse reactions of (**A**) leukocytes (%), (**B**) blood platelets (%) and (**C**) γ-glutamyl transpeptidase (%). *Indicates P < 0.05.

We observed the changes in values measured by routine blood, liver and kidney function tests, and found that adverse events regarding leucocytes, blood platelets and γ-glutamyl transpetidase (Fig. 5), NEUT, Hb, Tbil and AST (data not shown) were associated with the application cycle of the chemotherapy medicine. While these index levels increased during chemotherapy, they returned to normal values after cessation of treatment. During the 2^nd^ cycle of chemotherapy, compared to the control, TCM decreased the incidence of Hb reduction (11.9 *vs* 22.5%, *P* = 0.019) and the total bilirubin increase (42.1% *vs* 46.2%, *P* = 0.026) as well as blood platelet and γ-glutamyl transpetidase induced by chemotherapy. We have not seen the hematological toxicity for the long-term in subsequent chemotherapy cycles. To some extent, TCM caused the loss of appetite with more grade 1 or 2 toxic effects than found in the control group (*P* = 0.04).

## Discussion

Adjuvant chemotherapy is considered to be the gold standard after surgery for early stage patients with NSCLC, at least for stage II, resected IIIA, and selected IB NSCLCs^58^. Alternatively, platinum-based double adjuvant chemotherapy after surgery may be used to lower the risk of the recurrence of cancer. As shown in a LACE (Lung Adjuvant Cisplatin Evaluation) meta-analysis^59^, a cisplatin/vinorelbine combination was associated with a substantially superior survival benefit compared with other cisplatin-based regimens. However, toxicity has been a critical issue in platinum-based adjuvant protocols with neutropenia reported in up to 85% of patients and febrile neutropenia in up to 9% of treatments. About 50% of patients did not complete the entire adjuvant treatment due to toxicity^3^. Although the JRB.10 study indicated that the negative effects of adjuvant chemotherapy on QOL appeared to be temporary^56^, 75% of patients experienced grade 3 or 4 toxicity and 77% had at least one dose reduction or omission in a previous study^60^.

Symptoms such as loss of appetite, fatigue, pain and dyspnea reduce the QOL the most^61^. Furthermore, other studies have shown that the more symptoms of a disease, the lower the QOL is^62^,^63^,^64^. Increasing fatigue, dyspnea, cough and pain reduce the emotional dimension of QOL, while problems with night rest inevitably impair cognitive functioning^65^.

TCM has been successfully applied to postoperative lung cancer patients, with the special advantages of a reduction in tumor recurrence and metastasis^66^,^67^, and an enhanced QOL^68^, by alleviating symptoms such as shortness of breath, talk fatigue, coughing, loss of appetite and dried stools^69^. In addition, it has been reported that TCM combined with chemotherapy lessened the moderate to severe myelosuppression caused by conventional chemotherapeutic drugs^66^,^70^. According to previous meta-analysis of TCM when used as an adjunct therapy for NSCLC patients, including 24 studies of 2,109 patients, the frequently used herbs were Radix astragalus, Radix adenophorae, Radix ophiopogonis, Poria, Radix asparagi, Radix glycyrrhizae, Herba oldenlandia diffusa, Semen persicae and Radix notoginseng, from which the first 5 were also part of our formulations; Radix astragalus as well as Radix ophiopogonis are commonly used in the treatment of NSCLC in China^71^.

There are no published prospective, double-blinded trials of a TCM plus NP (vinorelbine plus platinum-based) regimen following surgical resection of NSCLC. By comparing the adjuvant chemotherapy (AC) with the placebo, the results of our study conclusively showed that the addition of TCM to AC provides better QOL scores for completely resected early stage NSCLC patients compared to chemotherapy agents alone.

In our study, role functioning impairment is usual for individuals after surgery and cognitive functioning is the main impairment after AC. TCM not only improved the scores of role functioning but also improved the cognitive function of patients after the 4 cycles of AC. Worry about future and symptom scores, such as fatigue, pain and dyspnea were high at baseline in both groups, but TCM released patients from pain, coughing blood, and pain in the chest. The combined treatment was significantly better compared with those patients who received 4 cycles of solely chemotherapy. TCM combined with chemotherapy was also effective in alleviating adverse events such as fatigue, diarrhea, dry mouth and vomiting. In our study, the incidence rate of adverse events was 0.57% in the intervention group, which was lower than in the control group (4.02%), a finding that was statistically significant (*P* = 0.037). A total of 8 patients terminated their treatment due to severe side effects, among which 7 were in the control group and only 1 patient in the intervention group.

It can be concluded that TCM combined with chemotherapy is safe. During the 2^nd^ cycle of chemotherapy, TCM plus chemotherapy increased the incidence of hematological hemoglobin reduction and the total bilirubin increase compared to chemotherapy alone. We have not yet seen the long-term hematological toxicity data for subsequent chemotherapy cycles.

Our clinical trial began enrollment from December 14, 2012, with the last patient being enrolled on March 26, 2015, and the last follow-up performed on August 1, 2015. By now, the longest follow-up time is 39.6 months, but further follow-ups will be required to confirm the survival benefit of TCM plus AC for resected lung cancer.

In summary, this study has shown that TCM can improve the temporary negative effects of AC on QOL and alleviate the symptoms with good safety. The results of this study provide additional practice guidelines and justification for the administration of TCM as an adjuvant chemotherapy in resected early stage NSCLC patients^72^,^73^,^74^,^75^,^76^,^77^,^78^,^79^,^80^.

## Additional Information

**How to cite this article**: Jiao, L. *et al*. Effects of Chinese Medicine as Adjunct Medication for Adjuvant Chemotherapy Treatments of Non-Small Cell Lung Cancer Patients. *Sci. Rep.* **7**, 46524; doi: 10.1038/srep46524 (2017).

**Publisher's note:** Springer Nature remains neutral with regard to jurisdictional claims in published maps and institutional affiliations.

## Supplementary Material

Supplementary Tables

## Figures and Tables

**Table 1:** Comparison of demographic data between the combined chemotherapy intervention group with Chinese traditional medicine and the chemotherapy alone control group before enrollment in the intention-to-treat population.

Characteristic	Intervention group (*n* = 156)	Control group (*n* = 158)	*P-*value
Age			
Median (years)	58.9 (39–77)	59.1 (36–78)	
<65 (%)	122 (78.21)	121 (76.58)	0.731
≥65 (%)	34 (21.79)	37 (23.42)	
Sex- *n* (%)			
Male	95 (60.90)	99 (62.66)	0.748
Female	61 (39.10)	59 (37.34)	
Disease stages at entry - *n* (%)			
IB	69 (44.23)	70 (44.30)	0.971
IIA	21 (13.46)	23 (14.56)	
IIB	15 (9.62)	13 (8.23)	
IIIA	51 (32.69)	52 (32.91)	
Histology - *n* (%)			
Squamous carcinoma	40 (25.64)	39 (24.68)	0.726
Adenocarcinoma	112 (71.79)	110 (69.62)	
Adenosquamous carcinoma	3 (1.92)	7 (4.43)	
Large cell carcinoma and others	1 (0.64)	2 (1.26)	
ECOG PS - *n* (%)			
0	2 (1.3)	3 (1.9)	0.330
1	152 (97.4)	155 (98.1)	
2	2 (1.3)	0 (0.00)	

Date are *n* (%) or median (range) for all patients with confirmed disease who received at least one cycle of the study drug. ECOG = Eastern Cooperative Oncology Group; PS = performance status.

**Table 2:** Adverse event rates *n* (%).

	Intervention group (*n* = 175)	Control group (*n* = 174)	*P*-value
At least one AE* (include SAE**)			
Yes	1 (0.57)^§^	7 (4.02) ※	<0.05
No	174 (99.43)	167 (95.98)	
At least one AE related to the medication (including SAE)			
Yes	0 (0.00)	3 (1.72)	0.123
No	175 (100.00)	171 (98.28)	
At least one SAE			
Yes	1 (100.00)	2 (28.57)	0.375
No	0 (0.00)	5 (71.43)	
At least one SAE related with medication			
Yes	0 (0.00)	1 (0.57)	0.499
No	175 (100.00)	173 (99.43)	
Drop out or stop trial because of AE & SAE			
Yes	1 (0.57)	6 (3.45)	0.067
No	174 (99.43)	168 (96.55)	
Death because of AE & SAE			
No	175 (100.00)	174 (100.00)	0.055

^*^AE: adverse event, **SAE: severe adverse event, ^§^AE = bronchopleural fistula (BPF), #x0203B; gastrointestinal tract reactions (*n* = 2), acquired pneumonia (n = 1), atrial fibrillation and heart disease (*n* = 1), hepatorenal toxicity (*n* = 1), fever chills (*n* = 1) and tuberculosis recurrence (*n* = 1).

**Table 3:** Adverse events at day 7 during the 1^st^, 2^nd^, 3^rd^ and 4^th^ cycle of chemotherapy.

Adverse Event	Intervention group				Control group				*P*-value
	*n*	Grade 0	Grade 1 or 2	Grade 3 or 4	*n*	Grade 0	Grade 1 or 2	Grade 3 or 4	
1^st^ cycle	128				129				
Pain		114 (89.1%)	14 (10.9%)	0		107 (76.98%)	32 (23.0%)	0	<0.05
2^nd^ cycle	120				124				
Hb		77 (64.20%)	16 (13.3%)	27 (22.50%)		107 (79.9%)	11 (8.2%)	16 (11.90%)	<0.05
Bilirubin		56 (46.7%)	5 (4.2%)	59 (49.20%)		84 (55.1%)	2 (2.8%)	48 (42.1%)	<0.05
Dry mouth		104 (86.7%)	15 (12.5%)	0		97 (72.4%)	37 (27.6%)	0	<0.01
3^rd^ cycle	114				126				
Vomiting		84 (73.7%)	27 (23.7%)	2 (1.8%)		71 (56.35%)	55 (43.7%)	0	<0.01
Diarrhea		112 (98.2%)	1 (0.9%)	0		116 (92.10)	10 (7.9%)	0	<0.05
4^th^ cycle	105				122				
Dry mouth		93 (88.6%)	10 (9.5%)	0		99 (81.1%)	23 (18.9%)	0	<0.05

Hb = Hemoglobin B.

**Table 4:** Adverse events at day 14 during the 1^st^, 2^nd^, 3^rd^ and 4^th^ cycle of chemotherapy.

Adverse Event	Intervention group (*n* = 107)			Control group (*n* = 122)			*P*-value
	Grade 0	Grade 1 or 2	Grade 3 or 4	Grade 0	Grade 1 or 2	Grade 3 or 4	
4^th^cycle							
Fatigue	75 (70.1%)	28 (26.2%)	0	77 (63.1%)	45 (36.9%)	0	<0.05
Diarrhea	103 (96.3%)	0 (0.0%)	0	117 (95.9%)	5 (4.10%)	0	<0.05
Loss of appetite	74 (69.2%)	29 (27.1%)	0	97 (79.5%)	25 (20.5%)	0	<0.05
Dry mouth	98 (91.6%)	5 (4.7%)	0	110 (90.2%)	12 (9.8%)	0	<0.05
