# Supplementary material for: Effects of Chinese Medicine as Adjunct Medication for Adjuvant Chemotherapy Treatments of Non-Small Cell Lung Cancer Patients
Source: Sci Rep. 2017 Apr 24;7:46524. doi: 10.1038/srep46524 (PMC5402288; doi:10.1038/srep46524)
Supplement: Supplementary Tables [file srep46524-s1.doc]

**Effects of Chinese Medicine as Adjunct Medication for Adjuvant Chemotherapy Treatments of Non-Small Cell Lung Cancer Patients**

Lijing Jiao^1,2*^, Changsheng Dong^1,2*^, Jiaxiang Liu^1,2^, Zhiwei Chen^3^, Lei Zhang^4^, Jianfang Xu^5^, Xiaoyong Shen^6^, Jiaming Che^7^, Yi Yang^8^, Hai Huang^9^, Hegen Li^1^, Jianli Sun^1^, Yi Jiang^1^, Zhujun Mao^1^, Peiqi Chen^2^,Yabin Gong^2^, Xiaolin Jin^2^, Ling Xu^1,2,10#^

^1^Department of Oncology, Longhua Hospital, Shanghai University of Traditional Chinese Medicine, Shanghai 200032, China

^2^Tumor Institute of Traditional Chinese Medicine, Shanghai Research Institute of Traditional Chinese Medicine, Shanghai 200032, China

^3^Lung Tumor Clinical Medical Center, Shanghai Chest Hospital, Shanghai Jiaotong University, Shanghai 200030, China

^4^Departmentof Thoracic surgery, Shanghai Pulmonary Hospital, Tongji University, Shanghai 200433, China

^5^Departmentof Oncology, Shanghai Pulmonary Hospital, Tongji University, Shanghai 200433, China

^6^Department of Thoracic Surgery, Huadong Hospital, Fudan University, Shanghai 200040, China

^7^Department of Thoracic Surgery, Ruijin Hospital, Shanghai Jiaotong University, Shanghai 200025, China

^8^Department of Thoracic Surgery, Shanghai Sixth People’s Hospital, Shanghai Jiaotong University, Shanghai 200030, China

^9^ Department of Pneumology, Changzheng Hospital, The Second Military Medical University, Shanghai 200003, China

^10^Department of Oncology, Yueyang Hospital of Integrated Traditional Chinese and Western Medicine, Shanghai University of Traditional Chinese Medicine, Shanghai 200437, China

*Made equal contributions to the research

**^#^Corresponding author:**

**Ling Xu, MD**

Department of Oncology, Yueyang Hospital of Integrated Traditional Chinese and Western Medicine, Shanghai University of Traditional Chinese Medicine, Shanghai 200437, China

Department of Oncology, Longhua Hospital, Shanghai University of Traditional Chinese Medicine. No. 725 South Wanping Road, Shanghai 200032, China

Email: nallclonghua@aliyun.com

Phone:86-21-64385700-9602

Fax: 86-21-64398310

Supplementary Table 1: Ingredients of formula I

| Plant name | Produced from | Biological/Pharmacological Activity | Active Components |
| --- | --- | --- | --- |
| Astragalus | Dry rhizoma | Anti-fatigue, exercise-induced anti-fatigue ^13,14^ | Astragalus flavonoids ^13,14^ |
| Codonopsis pilosula | Dry rhizoma | Immune modulatory ^15^  Anti-tumor ^16^  Anti gut damage ^17^ | Polysaccharides, saponins, sesquiterpenes, polyphenolic glycosides, alkaloids, polyacetylenes, essential oils and phytosteroids ^72^ |
| Atractylodes macrocephala | Dry rhizoma | Inhibit the ovalbumin (OVA)-mediated allergic diarrhea stimulation of the Th1-type immune responses ^18^ | Atractylodes macrophala koidz polysaccharides |
| Poria cocos | Dry sclerotia | Antioxidant ^19^ | Triterpenes ^73^ |
| Epimedium | Herba | Antioxidant ^74^ | Phenolic Compounds ^74^ |
| Trigonella foenum-graecum | Dry seed | Antioxidant ^20^  Enhanced endurance by utilization of fatty acids as an energy source ^21^  Ameliorates various impairments associated with physical fatigue ^22^ | Isoleucine polyphenol, flavonoid and amino acid contents ^22^ |
| Psoralea corylifolia L. | Fruit | Antidepressant, inhibits monoamine oxidase A and B (MAO-A and MAO-B) activities ^23^ | Coumarin, flavonoids, terpene phenolic ^75^ |

Supplementary Table 2: Ingredients of formula II

| Plant name | Produced from | Biological/Pharmacological Activity | Active Components |
| --- | --- | --- | --- |
| Radix adenophorae | Dry rhizoma | Suppressed development on inflammation and decreased airway damage by suppressing to T cells activity, eosinophilia, and bronchial hyperresponsiveness ^24^ | Polysaccharides, β-sitosterin, triterpenes |
| Glehnia litoralis | Dry rhizoma | Anti-cancer ^25^ | coumarins, triterpenes, volatile oil, polysaccharides |
| Radix asparagi | Dry rhizoma | Stimulation of salivary secretion Xerostomia ^26^ | furostanol saponins, β-sitosterin |
| Ophiopogon japonicus | Dry rhizoma | Stimulation of salivary secretion ^26^  Antioxidant and immunoregulatory ^27^ | Homoisoflavonoids ^76^ |
| Lilium brownii | Scale leaf | Antitumor ^28^  Anti-fatigue ^29^ | Lily polysaccharide ^28^ |
| Ligustrum lucidum | Fruit | Proliferation inhibition and apoptosis induction of cancer cells ^30-33^ | Ursolic acid |

Supplementary table 3: Ingredients of formula IV

| Plant name | Produced from | Biological/Pharmacological Activity | Active Components |
| --- | --- | --- | --- |
| Prunella vulgaris L. | Dry spikes | Antitumor ^34^  Stimulates macrophage activation ^35^  Enhances apoptosis ^36^ | Rosmarinic, ellagic and caffeic acids ^77^  Phenolic acids, flavonoids, coumarins, triterpenes, volatile oil, polysaccharides ^34,36^ |
| Arisaema heterophyllum Blume | Dry rhizoma | Antitumor ,apoptosis and autophagy ^37^ | Glycerol monostearic acid, ß-sitosterol, daucosterol and succinic acid ^78^ |
| Rhizoma amorphophalli | Dry tubers | Antitumor ^38^ | Konjac glucomannan ^38^ |
| Cremastra appendiculata | Dry pseudobulb | Antitumor ^39^ | Biphenanthrenes ^39^ |
| Seaweed | Leaves | Antitumor and antioxidant ^41^ ^40^ | Polysaccharides ^41^, lipids, pigment, polyphenols and terpenoids |
| Fructus trichosanthis | Pericarp | Antitumor ^42^ | Quercetin ^42^ |
| Ranunculus ternatus | Herba | Antitumor ^43^ ^44^ | Saponins, polysaccharides ^43^ ^44^ |
| Euphorbia helioscopia. | Herba | Antitumor ^45^ | Jatrophane-type diterpenoids ^79^ |
| Selaginella doederleinii Hieron | Herba | Antitumor, anti-inflammatory, anti-oxidant, anti-fungal and anti-virus activity ^48^ | Biflavonoids ^48^ |
| Salvia chinensis Benth. | Herba | Antitumor ^46^ | Total flavonoids ^80^ |
| Paris polyphylla | Dry rhizoma | Antitumor ^47^ | Polyphyllin VII ^47^ |
| Oyster shell | Shell | Sedative and hypnotic, sedative ^49^ | CaCO3 |
| Jujube date | Fruit | stimulated erythropoietin ^50^  antioxidative, antitumor ^51^  anti-fatigue ^52^ | Polysaccharides ^52^ |
